# Dynamic Ochratoxin A Production by Strains of *Aspergillus niger* Intended Used in Food Industry of China

**DOI:** 10.3390/toxins11020122

**Published:** 2019-02-18

**Authors:** Xiaomin Han, Hongru Jiang, Fengqin Li

**Affiliations:** 1 NHC Key Laboratory of Food Safety Risk Assessment, China National Center for Food Safety Risk Assessment, Beijing 100021, China; hanxiaomin@cfsa.net.cn; 2 National Institute for Nutrition and Health, Chinese Center for Disease Control and Prevention, Beijing 10050, China; jianghr@ninh.chinacdc.cn

**Keywords:** *Aspergillus niger*, Ochratoxin A, Food industry, China

## Abstract

Thirty strains of *Aspergillus niger*, including 27 intended used in the food industry of China, were studied for their ochratoxin A (OTA) production on the three natural substrates—corn, rice, and wheat bran—at different time intervals by high-performance liquid chromatography. It was found that the frequencies of OTA for the studied 27 industrial strains ranged from 14.8% (4/27) at day 28 to 25.9% (7/27) at day 7 on corn, 14.8% (4/27) at day 7 to 33.3% (9/27) at day 21 on rice, and 22.2% (6/27) at day 7, 14, and 28 to 44.4% (12/27) at day 21 on wheat bran, respectively. The average concentrations of OTA produced by the studied 27 industrial strains ranged from 5.1 μg/kg at day 28 to 8.7 μg/kg at day 21 on corn, 4.2 μg/kg at day 7 to 17.9 μg/kg at day 14 on rice, and 4.5 μg/kg at day 7 to 7.2 μg/kg at day 21 on wheat bran, respectively. Furthermore, the OTA production in the studied 27 industrial strains of *A.niger* was strongly associated with their function (or application), culture substrate, and time. The saccharifying enzyme producers produced higher levels of OTA, compared with the organic acid producers, the tannase producers, and the β-galactosidase producer, while concentration differences were also observed in OTA production among strains of *A.niger* with the same application. In a word, some strains of *A.niger* intended used in the Chinese food industry indeed have the capability of producing OTA, elevating the risks to food safety associated with their use.

## 1. Introduction

Ochratoxin A (OTA) was originally described as a metabolite of *Aspergillus ochraceus* by Wilhem in 1965, and the following studies indicated that many kinds of fungal species, including *Penicillium* such as *P.expansum*, *P.verrucosum*, and *P.chrysogenum*, as well as *Aspergillus* such as *A.niger*, *A.melleus*, and *A.albertensis*, were able to produce ochratoxins [1,2]. OTA possesses nephrotoxic, immunosuppressive, teratogenic, and carcinogenic properties, and was listed as a Group 2B carcinogen by the International Agency for Research on Cancer (IRAC) in 1993 [3,4].

*Aspergillus niger*, as one of the most important industrial filamentous fungal species, is employed in biotechnology and is also one of the most common natural contamination fungi in food and feed [5,6,7]. Although it was regarded as Generally Regarded As Safe (GRAS) by the U.S. Food and Drug Administration (FDA) in 1987, several reports revealed that strains of *A.niger* isolated from several substrates such as raisins, grapes, maize, mixed feeds and component raw materials, and Colombian coffee beans could produce OTA [6,7,8,9,10]. However, only a few reports were about the production of OTA in strains of *A.niger* used in the food industry [11,12,13], and there were no reports about the OTA production for strains of *A.niger* used in the food industry of China. Therefore, our aim is to establish the OTA temporal producing profile on different natural substrates for strains of *A.niger* intended for use in the food industry of China in this study.

## 2. Results

### 2.1. OTA Temporal Producing Profile for Strains of A.niger Used in This Study

Thirty strains of *A.niger* were used to survey their dynamic OTA producing profiles (Table 1). Firstly, it should be noted that the culture conditions of OTA production for the strains of *A.niger* used in this study were the same as those of the fumonisin (FB) production which we reported previously [14]. It was found that the positive rates of OTA produced by the studied 30 strains ranged from 13.3% at day 28 to 26.7% at day 7 and day 21 on corn, 13.3% at day 7 to 30.0% at day 21 on rice, and 20.0% at day 14 and day 28 to 40.0% at day 21 on wheat bran, while the average concentrations of OTA for those strains ranged from 4.6 μg/kg at day 28 to 7.8 μg/kg at day 21 on corn, 3.8 μg/kg at day 7 to 16.2 μg/kg at day 14 on rice, and 4.1 μg/kg at day 7 to 6.5 μg/kg at day 21 on wheat bran, respectively. Therefore, the concentrations of OTA at different time intervals were far lower than those detected for FB_2_ under the same culture conditions [14]. The maximum levels of OTA for the studied 30 strains of *A.niger* at all time intervals were 181.5 μg/kg for corn, 427.8 μg/kg for rice, and 175.4 μg/kg for wheat bran, while the maximum levels of FB_2_ for those strains under the same culture conditions were 70,488 μg/kg for corn, 54,284 μg/kg for rice, and 38,094 μg/kg for wheat bran [14]. The positive rates and the concentration levels of OTA for the saccharifying enzyme producers were higher than those produced by the organic acid producers, the tannase producers, and the β-galactosidase producer, a result which corresponded with that of our previous report for FB_2_ production [14]. As with the FB_2_ production in those strains, concentration differences were also observed in OTA production among the strains with the same function. For example, the saccharifying enzyme producers coding SN-01 and SN-11 yielded OTAs at higher levels at all time intervals for corn (SN-01) and wheat bran (SN-11), with a minimum level of 90.3 μg/kg for SN-01 on corn and 75.9 μg/kg for SN-11 on wheat bran, compared to those yielded by the saccharifying enzyme producers coding SN-03, SN-08, SN-09, and SN-12 under the same culture conditions, in which no OTA was detected on any of the three studied substrates, with the exception of SN-08 on wheat bran at day 21 and SN-12 on rice at day 14. Seven tannase producers and six organic acid producers only yielded OTA on one or two substrates at all time intervals (maximum < 15.0 μg/kg), except for one tannase producer coding TA-07 at day 14 (concentration = 427.8 μg/kg), day 21 (concentration = 176.5 μg/kg), and day 28 (concentration = 190.2 μg/kg) on rice. For the β-galactosidase producer GA-01, it was negative for OTA on corn and wheat bran at all time intervals, but it was positive for OTA at low levels (maximum < 20 μg/kg) on rice at all time intervals. Strains of ACCC 30557 and ATCC 16404 did not produce OTA on any of the three natural substrates, with the exception of ACCC 30557 on wheat bran at day 7 (concentration = 0.6 μg/kg) and ATCC 16404 on corn at day 7 (concentration = 0.2 μg/kg) and day 21 (concentration = 0.3 μg/kg). One strain of *A.niger* isolated from corn in our lab that coding SI-01 was negative for OTA on all three studied substrates at all time intervals, with the exception of corn at day 21 (concentration = 0.3 μg/kg). 

### 2.2. Substrates Effect on OTA Production Profile by A.niger Intended Used in Food Industry

The OTA production profile for strains of *A. niger* with different functions on different substrates was presented in Table 2. First, it was found that the OTA production in 27 strains of *A.niger* varied by substrates for SN-01 (*p* < 0.01), SN-06 (*p* < 0.01), SN-11(*p* < 0.01), OA-06 (*p* < 0.01), and TA-07 ((*p* < 0.05). For the studied saccharifying enzyme producers, on corn, their average levels of OTA increased steadily and reached a maximum of 68.3 μg/kg at day 28, whereas on rice, their average concentrations of OTA decreased over time with a maximum of 28.5 μg/kg at day 7 and a minimum of 5.8 μg/kg at day 28; however, on wheat bran, the average concentration of OTA increased until day 14 after inoculation with a maximum of 52.3 μg/kg; then, it decreased to an average of 25.4 μg/kg at day 21 and increased again to an average concentration of 27.4 μg/kg at day 28. Hence, high average concentrations of OTA on corn and wheat bran were observed in comparison with those on rice except on day 7 for thirteen saccharifying enzyme producers. Regarding the six organic acid producers, at least one strain was positive for OTA in corn at all time intervals, but no OTA was detected on rice on day 7 and day 14 and on wheat bran on day 7 and day 28. On the other hand, for the seven tannase producers, at least two strains yielded OTA on wheat bran at all time intervals, and only one strain was positive for OTA on day 21 on corn, and only one or two strains were positive for OTA at day 14, 21, and 28 on rice, respectively. For instance, one tannase producer coding TA-07 produced high levels of OTA on rice on day 14, 21, and 28 (concentration = 427.8 μg/kg, 176.5 μg/kg, and 190.2 μg/kg), but yielded low levels of OTA (or none) on corn and wheat bran. For one β-galactosidase producer, only 0.2 μg/kg and 15.9 μg/kg of OTA were detected on rice on day 21 and day 28, respectively. Furthermore, lower positive rates and average concentrations of OTA for the strains of *A.niger* with the same function on the studied three substrates were observed than those detected for FB_2_ [14]. For instance, 100% of the saccharifying enzyme producers produced FB_2_ on all three studied natural substrates at all time intervals, while only 40% or less yielded OTA on the studied three substrates at all time intervals except for those strains on day 7 on corn (positive = 46%) and at day 21 on wheat bran (positive = 54%). In addition, only one or two organic acid producers were positive for OTA on the studied three substrates, with lower than average concentrations of OTA (maximum = 7.4 μg/kg), which was also far lower than those for FB_2_ [14]. This observation pointed to the important effect of the components of the substrates on OTA production for *A.niger* and its intended uses in the Chinese food industry, and it could be concluded that higher levels of OTA were usually produced on wheat bran and corn than those detected on rice under the same culture conditions.

### 2.3. Average Concentration Distribution of OTA Produced by 27 Strains of A.niger Intended Used in Chinese Food Industry

**Figure 1:**
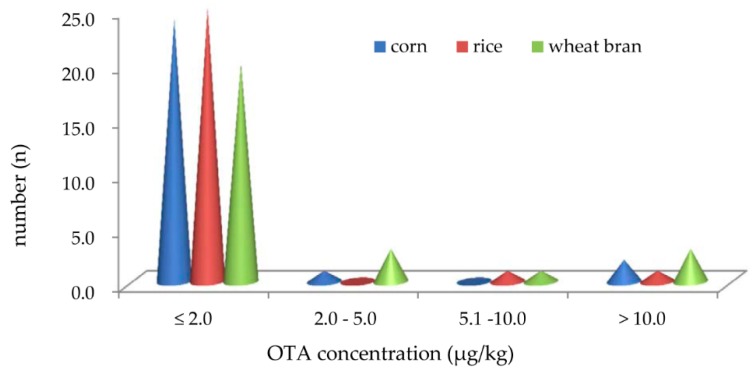
OTA average concentration distribution produced by 27 strains of *a.niger* with intended uses in the Chinese food industry. *n* = OTA positive strain number.

The average concentration distribution of OTA produced by 27 strains of *A.niger* at all time intervals on the three natural substrates is given in Figure 1. According to the national food safety standard, Maximum Levels of Mycotoxins in Foods GB2761-2017 in China, the maximum levels of OTA in grape wine, coffee beans and coffee powder, and instant coffee were 2.0 μg/kg, 5.0 μg/kg, and 10.0 μg/kg, respectively. Therefore, the 27 strains tested were separated into four groups, and they were ≤2.0 µg/kg, 2.0–5.0 µg/kg, 5.1–10.0 µg/kg, and >10.0 µg/kg based on their OTA average levels at all time intervals on different substrates. Two (2/27, 7%) strains on corn, one (1/27, 4%) strain on rice, and three (3/27, 11%) strains on wheat bran produced OTA at an average concentration higher than 10.0 µg/kg, with a maximum of 129.3 μg/kg on corn, 264.8 μg/kg on rice, and 115.7 μg/kg on wheat bran, respectively, while there was one (1/27, 4%) strain producing OTA at an average concentration between 5.1 µg/kg and 10.0 µg/kg on rice and wheat bran, but none on corn. Only one (1/27, 4%), zero (0/27, 0%), and three (3/27, 11%) strains yielded the average levels of OTA, ranging from 2.0 µg/kg to 5.0 µg/kg on corn, rice, and wheat bran, respectively. Almost all strains, including 24 (89%) on corn, 25 (93%) on rice, and 20 (74%) on wheat bran, synthesized OTA at average levels below or equal to 2.0 µg/kg on the three natural substrates. Therefore, the highest yield of OTA was produced on wheat bran and corn, followed by that on rice, in a trend similar to that for FB_2_ production, as reported earlier [14].

## 3. Discussion

In food and feed applications, strains of *A. niger*—representing the workhorses of the industrial biotechnology—have a long and extensively documented history of safe use [13]. Approximately 1–41% strains of *A.niger* isolated from foods such as dried vine fruits and wine grapes were capable of producing OTA depending on their commodity and their culture conditions [9,10,11,15,16]. Frisvad et al. reported that among the industrial strains of *A.niger* (*n* = 69), about 33% of them could produce OTA, including the three important industrial strains NRRL 337, NRRL 3112, and NRRL 3122 [11]. To the best of our knowledge, this is the first report on the OTA production by strains of *A.niger* with intended uses in the Chinese food industry. The positive rates of OTA for 27 strains of *A.niger* used in our study ranged from 14.8% (4/27) on corn and rice to 44.4% (12/27) on wheat bran, which included the OTA frequency for the industrial strains of *A. niger* (positive rate = 33%), but higher than those for the nonindustrial strains of *A.niger* (positive rate = 7%) reported by Frisvad et al. [11]. All these differences may result from the genetic variations (containing or not containing the toxin-producing genes) or from the culture condition differences, especially the culture temperature, the time, and the medium [9,10,11,15,16,17].

Interestingly, the positive rates and the concentrations of OTA produced by strains of *A.niger* used in this study were highly related to their application. Generally, strains used for saccharifying enzyme production yielded higher levels of OTA than those used for the organic acid production and the tannase production under the same culture conditions. In addition, strains of *A.niger* with the same function yielded different amounts of OTA under the same culture conditions. Among the thirteen saccharifying enzyme producers, strains coding SN-01 and SN-011 were high OTA producers, while strains coding SN-03, SN-08, SN-09, and SN-12 were low OTA producers or nonproducers. In order to clarify these differences, it was necessary to simultaneously detect their OTA concentrations and their related products, such as enzymes and organic acid [18,19,20,21,22,23], as well as the related genes and their expressions which participated in OTA biosynthesis [11,12].

Last, the positive rates and the average concentrations of OTA in the studied strains of *A.niger* were very low compared with the positive rates and the average concentrations of FB_2_ produced by strains of *A.niger* under the same conditions, which we have reported previously. All the concentrations of OTA were in the levels of μg/kg (maximum = 427.8 μg/kg), while most of the concentrations of FB_2_ were in the levels of mg/kg (maximum = 70,488 μg/kg) [14]. Because the culture conditions for FB_2_ production and OTA production were exactly same, the only differences were the different toxin detection methods. It was inferred that there was no correlation between the FB_2_ production and the OTA production, which was in line with the reports by Frisvad et al. and Susca et al. [11,12].

Because of their potential mycotoxin production and pathogenicity to humans and animals, strains of *A.niger* have been considered as risk group 2 biological agents by the German Federal Institute for Occupational Safety and Health [13]. Our findings show that strains of *A.niger* intended used in the Chinese food industry are indeed capable of producing OTA, and their toxin-producing abilities are highly related to their industrial applications and their culture conditions such as time and substrate, as well as being strain-specific. Because strains of *A.niger* are one of the most important industrial fungi for food additive production in China, it is very important to evaluate their toxin-producing ability before industrial use.

## 4. Materials and Methods

### 4.1. Chemicals and Reagents

A standard of OTA with purities higher than 98% was purchased from Romer Labs (IFA-Tulln, Austria). All the organic solvents such as acetic acid, methanol, and acetonitrile used in this study in the HPLC grade were obtained from Fisher Scientific (Fair Lawn, NJ, USA). Other reagents such as NaCl, NaHCO_3_, and tween 20 were of analytical grade and obtained from Sigma-Aldrich (St. Louis, MO, USA). An OchraTest immunoaffinity column was purchased from VICAM (Clover Technology Group, Beijing, China). Pure water was prepared in our lab by a Millipore Milli-Q system (Millipore, Bedford, MA, USA) with a conductivity higher than 18.2 MΩ at 25 °C.

### 4.2. Strains of Fungi

There were thirty strains of *A.niger* used in this study. From the point of view of the industrial application, the twenty-seven strains of *A.niger* with intended uses in the Chinese food industry were divided into four groups, including the thirteen strains used for saccharifying enzyme production, the six strains used for organic acid production, the seven strains used for tannase production, and one strain used as for β-galactosidase production. In addition, there were three strains where one was used for research and teaching in classification, another used for antifungal activity determination, and one strain isolated from corn. Detailed information about the thirty strains of *A.niger* is shown in Table 3.

### 4.3. OTA Production

For the activation and sporulation of the strains of *A.niger*, the studied thirty strains were inoculated on the Czapek agar slants, respectively, and incubated at 28 ± 1 °C for one week. The spore suspension of *A.niger* was made by using sterilized distilled water, scraping the spores, and mixing thoroughly with the inoculating hook. Each flask containing 100 g polished rice or corn or wheat bran was autoclaved twice on successive days at 121 °C for 20 min. Before inoculation, the autoclaved flask containing rice or corn or wheat bran was adjusted to 20% (30% for wheat bran) relative moisture by adding sterilized distilled water, and then it was inoculated with 5 mL of one week old *A.niger* spore suspension or 5 mL sterilized distilled water used as a control in two parallel, respectively. After inoculation, all of them were incubated in the dark at 28 ± 1 °C for 4 weeks. For the time profile study, we collected 10 g of fermented cultures on day 7, day 14, day 21, and day 28 and detected the concentrations of OTA. All fermented cultures were left standing for the first three days after inoculation and shaken two times per day to reduce clumping. Three substrates—corn, rice, and wheat bran—without OTA or with OTA lower than the limit of detection were used as control.

### 4.4. OTA Extraction

The OTA extraction was performed based on the Chinese food safety standard GB/T 23502-2009: determination of ochratoxin A in food high-performance liquid chromatographic method with OchraTest immunoaffinity column clean-up with some modifications [23]. Briefly, 5.0 g of fermented cultures were homogenized completely by adding 20 mL of methanol–water (80:20, *v*/*v*), and extracted at 200 rpm from an orbital shaker (Eyela Inc., Tokyo, Japan) for 60 min, and the mixture was then centrifuged at 10,000 rpm for 15 min (Beckman Coulter, Brea, CA, USA). Ten mL supernatant was diluted with 40 mL phosphate buffer (8.0 g NaCl, 1.2 g sodium hydrogen phosphate, 0.2 g potassium dihydrogen phosphate, and 0.2 g potassium chloride per 1L, pH 7.0). All the diluted extracts were pushed through the immunoaffinity column, followed by washing with 10 mL mycotoxin cleaning buffer (2.5 g NaCl, 5.0 g NaHCO_3_, and 0.1 mL Tween 20 per 1L) and 10 mL water. Finally, 1.5 mL methanol was used to elute the column, and the purified product was used for HPLC analysis.

### 4.5. HPLC Conditions

Detection and quantification of OTA were performed on an Agilent HPLC 1200 system with a fluorescence detector (Santa Clara, CA, United States) and an Agilent ZORBAX SB-C_18_ column (150 mm × 4.6 mm i.d., 5 μm particle size) at a temperature of 40 °C. It was detected in the isometric elution with the mobile phase and included acetonitrile as solvent A and 2.5% formic acid in water as solvent B (50:50, *v*/*v*) and a flow rate of 0.8 mL/min. In addition, the detecting excitation wavelength and the emission wavelength for the OTA detection were 333 nm and 477 nm, respectively.

### 4.6. HPLC Method Validation

Mean recoveries for the three substrates were detected with the three parallel analyses of OTA-free corn, rice, and wheat bran samples spiked with 1.0 μg/kg to 10.0 μg/kg, and they were in the range between 79.10 ± 10.31% and 102.60 ± 9.41% for corn, 80.80 ± 9.12% and 95.78 ± 6.71% for rice, and 83.13 ± 7.45% and 110.45 ± 6.45% for wheat bran, respectively. Method repeatability and reproducibility were also determined with the toxin-free samples by spiking OTA into the OTA-free substrates according to the requirements of European Union Decision 2002/657/EEC [24]. All these data shown that the relative standard deviations for OTA detection in the above three natural substrates ranged from 4.31% to 6.74% for intra-day and 5.89% to 10.65% for inter-day, respectively. The limit of detection and the limit of quantification for OTA detection in the studied three substrates were all 0.03 μg/kg and 0.1 μg/kg.

### 4.7. Data Analysis

All the concentrations of OTA at different time intervals on different substrates were obtained using the software OpenLAB CDS A.02.01 (Santa Clara, CA, USA). Parameters such as the frequency, the mean, and the range of OTA for strains of *A.niger* used in this study were obtained by applying the SPSS statistical package (version 20.0, IBM, Amund, NY, USA). The analysis of variance (ANOVA) was employed for statistical analysis by comparing the concentrations of OTA on different substrates and time intervals.

## Figures and Tables

**Table 1:** Concentrations of ochratoxin A (OTA) for the strains of *A.niger* used in this study at different time intervals.

Stain No ^1^	Corn (μg/kg)				Rice (μg/kg)				Wheat Bran (μg/kg)			
	Day 7	Day 14	Day 21	Day 28	Day 7	Day 14	Day 21	Day 28	Day 7	Day 14	Day 21	Day 28
SN-01	128.0	117.4	181.5	90.3	nd	nd	nd	nd	nd	nd	0.1	nd
SN-02	1.3	0.3	nd	nd	0.8	0.4	0.2	0.2	2.0	nd	0.4	3.4
SN-03	nd ^2^	nd	nd	nd	nd	nd	nd	nd	nd	nd	nd	nd
SN-04	3.5	0.4	0.1	nd	0.1	nd	nd	nd	35.0	0.5	0.3	0.6
SN-05	4.7	21.8	52.0	46.2	88.0	29.0	22.3	14.8	nd	nd	nd	nd
SN-06	nd	nd	0.2	nd	25.0	26.0	9.8	8.0	nd	nd	nd	nd
SN-07	nd	nd	nd	nd	nd	nd	0.2	nd	5.0	nd	0.5	nd
SN-08	nd	nd	nd	nd	nd	nd	nd	nd	nd	nd	0.3	nd
SN-09	nd	nd	nd	nd	nd	nd	nd	nd	nd	nd	nd	nd
SN-10	2.0	nd	nd	nd	nd	nd	nd	nd	nd	nd	nd	nd
SN-11	0.2	4.1	nd	nd	nd	0.7	0.9	0.2	75.9	116.2	175.4	95.3
SN-12	nd	nd	nd	nd	nd	0.5	nd	nd	nd	nd	nd	nd
SN-13	nd	nd	nd	nd	nd	nd	nd	nd	nd	40.1	0.5	10.4
OA-01	nd	nd	nd	0.7	nd	nd	0.2	nd	nd	nd	13.2	nd
OA-02	nd	nd	nd	nd	nd	nd	nd	nd	nd	0.9	1.5	nd
OA-03	nd	nd	nd	nd	nd	nd	nd	nd	nd	nd	nd	nd
OA-04	nd	nd	nd	nd	nd	nd	nd	nd	nd	nd	nd	nd
OA-05	nd	nd	nd	0.5	nd	nd	0.8	0.2	nd	nd	nd	nd
OA-06	0.4	0.4	0.3	nd	nd	nd	nd	nd	nd	nd	nd	nd
TA-01	nd	nd	nd	nd	nd	nd	nd	nd	nd	nd	nd	nd
TA-02	nd	nd	nd	nd	nd	nd	nd	nd	nd	nd	nd	nd
TA-03	nd	nd	nd	nd	nd	nd	nd	nd	nd	nd	nd	nd
TA-04	nd	nd	nd	nd	nd	nd	nd	nd	1.9	1.9	1.0	11.7
TA-05	nd	nd	nd	nd	nd	0.2	nd	nd	nd	nd	nd	nd
TA-06	nd	nd	nd	nd	nd	nd	nd	nd	nd	nd	0.4	nd
TA-07	nd	nd	0.6	nd	nd	427.8	176.5	190.2	3.0	0.3	0.5	5.1
GA-01	nd	nd	nd	nd	nd	nd	0.2	15.9	nd	nd	nd	nd
ACCC 30557	nd	nd	nd	nd	nd	nd	nd	nd	0.6	nd	nd	nd
ATCC 16404	0.2	nd	0.3	nd	nd	nd	nd	nd	nd	nd	nd	nd
SI-01	nd	nd	0.3	nd	nd	nd	nd	nd	nd	nd	nd	nd

^1^ SN 01 to SN-13: saccharifying enzyme producer (*n* = 13); OA-01 to OA-06: organic acid producer (*n* = 6); TA-01 to TA-07: tannase producer (*n* = 7); GA-01: β-galactosidase producer (*n* = 1); SI-0: isolated from corn (*n* = 1). ^2^ nd: not detected or lower than the limit of detection (< LOD).

**Table 2:** Substrates effect on the frequencies and the mean concentrations of OTA produced by 27 strains of *A.niger* used as different applications.

Substrate	Time Intervals (Day)	OTA Production for Different Functions of *A.niger*					
		Saccharifying Enzyme Producer (*n* = 13)		Organic Acid Producer (*n* = 6)		Tannase Producer (*n* = 7)	
		No. of Positive (%)	Mean (Range) (μg/kg)	No. of Positive (%)	Mean (Range) (μg/kg)	No. of Positive (%)	Mean (Range) (μg/kg)
Corn	7	6 (46)	23.3 (0.2–128.0)	1 (17)	0.4	0	0
	14	5 (38)	28.8 (0.3–117.4)	1 (17)	0.4	0	0
	21	4 (31)	58.5 (0.1–181.5)	1(17)	0.3	1 (14)	0.6
	28	2 (15)	68.3 (46.2–90.3)	2 (33)	0.6 (0.5–0.7)	0	0
Rice	7	4 (31)	28.5 (0.1–88.0)	0	0	0	0
	14	5 (38)	11.3 (0.4–29.0)	0	0	2 (29)	214.0 (0.2–427.8)
	21	5 (38)	6.7 (0.2–22.3)	2 (33)	0.5 (0.2–0.8)	1 (14)	176.5
	28	4 (31)	5.8 (0.2–14.8)	1 (17)	0.2	1 (14)	190.2
Wheat bran	7	4 (31)	29.5 (2.0–75.9)	0	0	2 (29)	2.5 (1.9–3.0)
	14	3 (23)	52.3 (0.5–116.2)	1 (17)	0.9	2 (29)	1.1 (0.3–1.9)
	21	7 (54)	25.4 (0.1–175.4)	2 (33)	7.4 (1.5–13.2)	3 (43)	0.6 (0.4–1.0)
	28	4 (31)	27.4 (0.6–95.3)	0	0	2 (29)	8.4 (5.1–11.7)

**Table 3:** Information about the strains of *A.niger* used in this study.

Potential Application	Strain No.	Isolated Substrate	Optimum Culture Temperature (°C)	Obtained Center/Place ^2^
Saccharifying enzyme production (*n* = 13)	SN-01	nr ^1^	25–28	ACCC
	SN-02	Mold culture used for wine fermentation	25–28	ACCC
	SN-03	nr	25–28	ACCC
	SN-04	nr	25–28	ACCC
	SN-05	nr	30–32	ACCC
	SN-06	nr	30–32	ACCC
	SN-07	nr	25–28	CGMCC
	SN-08	nr	25–28	CGMCC
	SN-09	nr	25–28	CGMCC
	SN-10	nr	25–28	CGMCC
	SN-11	nr	25–28	CGMCC
	SN-12	nr	25–28	CGMCC
	SN-13	nr	25–28	CGMCC
Organic acid production (*n* = 6)	OA-01	nr	25–28	CGMCC
	OA-02	nr	25–28	CGMCC
	OA-03	nr	29–31	ACCC
	OA-04	nr	25–28	CGMCC
	OA-05	nr	25–28	CGMCC
	OA-06	nr	25–28	ACCC
Tannase production (*n* = 7)	TA-01	nr	25–28	CGMCC
	TA-02	Eucalyptus leaves	25–28	CGMCC
	TA-03	Rotten soil	25–28	CGMCC
	TA-04	nr	25–28	CGMCC
	TA-05	Rotten wood	25–28	CGMCC
	TA-06	Rotten wood	25–28	CGMCC
	TA-07	Rotten wood	25–28	CGMCC
β-galactosidase production (*n* = 1)	GA-01	Moldy bagasse	25–28	CGMCC
Research and Teaching	ACCC 30557	Cloth	24	ACCC
Used as the reference strain for medium control, method validation, etc.	ATCC 16404	nr	25–28	ATCC
No practical application	SI-01	Corn	25–28	Our lab

^1^ nr: no record; ^2^ ACCC: Agricultural Culture Collection of China, CGMCC: China General Microbiological Culture Collection Center, ATCC: American Type Culture Collection.
